# Melt Spinning of Highly Stretchable, Electrically Conductive Filament Yarns

**DOI:** 10.3390/polym13040590

**Published:** 2021-02-16

**Authors:** Henriette Probst, Konrad Katzer, Andreas Nocke, Rico Hickmann, Martina Zimmermann, Chokri Cherif

**Affiliations:** 1 Institute of Textile Machinery and High Performance Material Technology, TU Dresden, 01062 Dresden, Germany; andreas.nocke@tu-dresden.de (A.N.); rico.hickmann@tu-dresden.de (R.H.); chokri.cherif@tu-dresden.de (C.C.); 2 Fraunhofer Institute for Material and Beam Technology IWS, 01277 Dresden, Germany; konrad.katzer1@tu-dresden.de (K.K.); martina.zimmermann@tu-dresden.de (M.Z.); 3 Institute of Materials Science, TU Dresden, 01069 Dresden, Germany

**Keywords:** melt spinning, thermoplastic polyurethane (TPU), carbon nanotube (CNT), stretchable filament yarn, electrically conductive filament yarn

## Abstract

Electrically conductive fibers are required for various applications in modern textile technology, e.g., the manufacturing of smart textiles and fiber composite systems with textile-based sensor and actuator systems. According to the state of the art, fine copper wires, carbon rovings, or metallized filament yarns, which offer very good electrical conductivity but low mechanical elongation capabilities, are primarily used for this purpose. However, for applications requiring highly flexible textile structures, as, for example, in the case of wearable smart textiles and fiber elastomer composites, the development of electrically conductive, elastic yarns is of great importance. Therefore, highly stretchable thermoplastic polyurethane (TPU) was compounded with electrically conductive carbon nanotubes (CNTs) and subsequently melt spun. The melt spinning technology had to be modified for the processing of highly viscous TPU–CNT compounds with fill levels of up to 6 wt.% CNT. The optimal configuration was achieved at a CNT content of 5 wt.%, providing an electrical resistance of 110 Ωcm and an elongation at break of 400%.

## 1. Introduction

For numerous applications, e.g., in the field of smart textiles and textile sensor and actuator technology, electrically conductive fibers and filaments are of great importance. They are essential for the production of textile-processable sensors [1,2,3] and sensor networks [4] as well as for the transmission of information detected in the device. New developments in the smart textiles sector are inconceivable without electrically conductive fibers. For instance, they can transmit the data collected during wound monitoring [5,6] or mechanical structural health monitoring of critical components [7,8,9]. Furthermore, they are essential for the development of novel, wearable devices [10,11,12] and the storage of electrical energy [13].

In terms of actuator technology, the supplied electrical energy can be used to generate mechanical deformation. In shape memory alloys (SMA), the applied electrical energy combined with the intrinsic resistance of SMA causes temperature to increase, which in turn leads to a conversion in the crystal structure from martensite to austenite, thus generating large usable forces and strains [14,15,16,17]. In contrast, shape memory polymers do not have intrinsically conductive components, but they too are able to use electrical energy via the intermediate stage of thermal energy to perform mechanical work [18,19,20]. For this purpose, the entire component can either be exposed to an electric field or a constant temperature, or individual areas can be targeted separately. Electrically conductive yarns are particularly suited for the activation of individual textile parts.

According to the state of the art, metallized fibers, carbon fibers, or fine copper wires are commonly used to conduct electric current [21,22,23]. These materials feature very low electrical resistances as well as several specific disadvantages: metal-based conductors are generally characterized by a very low elongation at break and almost no elastic strain [24]. Previous studies showed that in the case of metal-coated fibers, the coating often peels off or breaks so that the electrical conductivity is significantly reduced, resulting in poor long-term stability.

Electrically conductive spinnable polymers are a viable option to overcome these disadvantages because—in comparison to metallized fibers—the physiological and mechanical properties of filled and unfilled polymer fibers exhibit reduced deviations. This ensures a high degree of structural and material compatibility. Furthermore, the functional component can be produced by means of a highly productive and automated spinning process without the need for additional work steps or production facilities. Moreover, spun filaments can achieve an elongation at break value that is significantly higher than that of metal-based yarns. Additionally, their electrical conductivity does not rely on a coating that is susceptible to mechanical damage, which is why they offer considerably enhanced durability.

The spinning of intrinsically electrically conductive polymers represents a great technical challenge; therefore, additives or fillers are often added to conventional spinning polymers to achieve electrical conductivity [24]. Trials have already been carried out on compounding polypropylene (PP) with carbon black (CB) [25,26], or PP with carbon nanotubes (CNTs) [27,28] and polyamide (PA) with CNTs [29,30]. The low mechanical elongations of PP and PA fibers make them unsuitable for numerous applications, especially in terms of actuator technology, where formability is a crucial criterion.

Further research activities deal with the production of electrically conductive composite yarns by using a CNT-based coating. For example, cotton threads could be coated with a CNT ink and provided with an ion-selective membrane. This made it possible to determine potassium, ammonium, and pH in human sweat, thus enabling the production of smart textiles that can monitor biological functions [31]. The goal of producing biochemical sensors was also pursued by Parrilla et al. who embedded commercial carbon fibers in a polymeric fiber matrix in order to determine the sodium concentration in sweat by means of textile-processable, wearable patches [32]. In addition, they succeeded in developing a CNT-based ink that could be applied to a polyurethane layer to perform multi-ion sweat analysis and could be used to coat conventional textiles [33,34]. Of particular note is the fact that the chemosensorial behavior of the CNT composite yarns is hardly affected by bending or stretching. This increases the fatigue strength of the biological sensors and allows them to be used in smart textiles that must follow the movements of a human wearer. However, this prevents them from serving as strain sensors. This goal, on the other hand, is being pursued by Li et al., who surrounded electrospun thermoplastic polyurethane (TPU) filaments with a CNT-based coating and used the resulting fibers as textile strain sensors. They achieved very good electrical conductivities of up to 13 S/cm [35]. Nevertheless, the manufacturing process based on electrospinning and coating has low productivity, which limits its use for the mass market. The present research work, on the other hand, aims at a highly productive, one-step manufacturing process for electrically conductive and highly stretchable filaments. For this purpose, thermoplastic polyurethane (TPU) was compounded with CNTs and processed by melt spinning.

TPUs are block copolymers consisting of hard and soft segments. The hard segments comprise a diisocyanate and a polyol, thus forming urethane groups (–NHCO–O–). In contrast, the soft segments consist of a polyester or polyether polyol [36,37]. The main difference between TPU and conventional elastomers is that in conventional elastomers, the cross-linking points generating material strength are formed by covalent bonds, whereas in thermoplastic elastomers such as TPU, they appear in the form of partially crystalline areas [38].

The soft segments have a glass transition point, which is below the usage temperature so that the molecules can be shifted flexibly due to low intermolecular interactions. Moreover, the soft segments exhibit an entropy-elastic behavior; i.e., the polymer chains that are entangled in a stress-free state are stretched under mechanical load with a decrease in entropy [39,40]. If the mechanical load is removed, the soft segments return to their energetically favorable initial state [41]. Thus, the soft segments cause the high elasticity of the polymer, whereas the hard segments determine the solid aggregate state at use temperature as well as the mechanical strength and stiffness [41]. The semi-crystalline hard segments in the TPU assume the function of covalent bonds (network points) in a conventional elastomer, hence preventing the polymer chains from gliding off against each other [37]. When TPU is heated, the intermolecular bonds between the hard segments are broken and the polymer becomes liquid so that it can be melt-spun. Thus, the structure of hard and soft segments enables thermal processing that is unsuitable for standard elastomers since they do not melt when heated but undergo decomposition processes.

In this study, the thermal processability of TPU is exploited to produce highly stretchable and electrically conductive multifilament yarns that can be used for a variety of tasks in the field of smart textiles and fiber elastomer composites. For this purpose, TPU is compounded with CNTs and melt spun. To enable a melt spinning process with this polymer material, which has a very high viscosity, a process modification is necessary to enable a particularly gentle drawing process. At filling levels of 5 wt.% CNT, electrical resistances of 110 Ωcm can be realized in the mechanically unloaded state. Even under relative mechanical strains of up to 100%, the electrical conductivity is maintained, but the electrical resistance increases by up to one order of magnitude.

## 2. Materials and Methods

For the melt spinning trials, the TPU grade Desmopan 9370A from Covestro AG (Leverkusen, Germany) [42] and TPU 1001 from Nanocyl SA (Sambreville, Belgium) [43] were used. TPU 1001 from Nanocyl SA is a masterbatch containing 10 wt.% CNT and 90 wt.% TPU. Before the spinning process was started, the materials were pre-compounded by hand to compounds of 1–6 wt.% CNT. The compounds were dried at a temperature of 80 °C for 24 h.

The tests were carried out on a bicomponent melt spinning plant of Dienes Apparatebau GmbH (Mühlheim am Main, Germany), at ITM, TU Dresden. This plant is equipped with a single-screw extruder, a twin-screw, and several spinning packages to realize different fiber geometries. The following tests were performed with a twin-screw extruder and a 60-filament core–sheath spinning die, although the extruder supplying the sheath component was not taken into operation. The 60-filament die has diameters of 0.6 mm. Each spinning process was performed with particularly coarse-meshed polymer filters and under a nitrogen atmosphere to avoid the oxidation of TPU. A spinning temperature of 180 °C was selected, and the winding speeds were varied between 8 and 650 m/min according to the compound’s spinnability.

**Figure 1:**
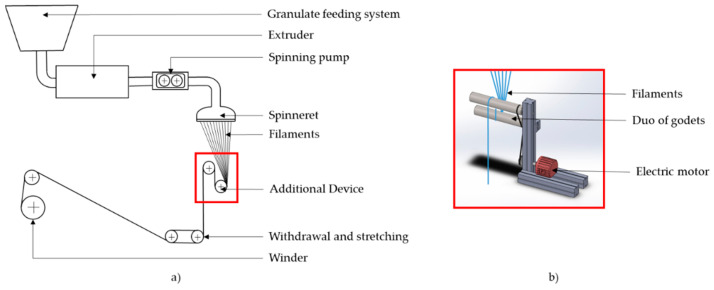
Additional godet device: (**a**) in operation; (**b**) as SolidWorks 3D model.

Extensive modifications to the spinning machine were required to ensure process stability. By means of an additional device, the weight of the solidified filaments was supported shortly below the spinneret so that the melt no longer had to support the entire weight of the filaments. For this purpose, a duo of godets driven by an electric motor was inserted into the spinning shaft 1 m below the spinneret (see Figure 1). Firstly, the spun filaments were guided over the lower cylinder, and, secondly, they ran vertically upwards while being drawn by the upper cylinder. Due to the staggered arrangement of the cylinders, further deflection points could be avoided to minimize potential effects on the yarn path, the geometry of individual filaments, and the arrangement of filaments in the fiber bundle. Once the spun filaments passed this additional device, they were taken off and wound up.

To determine the melt viscosity, rheometric measurements were performed on a Haake RheoWin /Thermo Scientific Mars II from Thermo Fisher Scientific Inc. (Waltham, MA, USA). The measurements were carried out at a constant temperature of 200 °C. The fineness was determined in accordance with DIN EN ISO 2060. For this purpose, 5 samples with a defined length of 1 m each were taken from each spinning specification. The mass of the samples was then determined using a precision scale R200D from Sartorius (Göttingen, Germany). The tensile tests were performed on a Zwicki Junior from ZwickRoell GmbH & Co. KG (Ulm, Germany) with a clamping length of 62.5 mm and a testing speed of 200 mm/min. Tensile testing as well as the determination of fineness were completed for 5 samples each.

**Figure 2:**
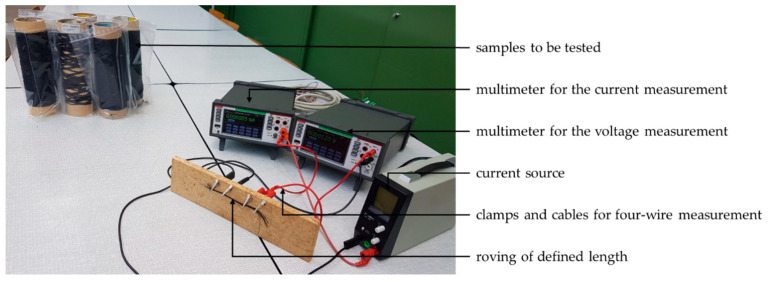
Test setup for the determination of electrical resistance.

A four wire method was employed for resistance measurements on filament sections with a length of 50 mm (see Figure 2). Additionally, a current source Voltcraft LRP-1601 (Wollerau, Switzerland) and two Keithley DAQ6510-7700 multimeters from Keithley Instruments Corp. (Solon, OH, USA) were used. The current source supplied a maximum current of 100 mA. For each sample, four different current values were set at the current source, and the multimeters were used to measure current and voltage at the clamped sample. Thus, four resistance values could be calculated and averaged for each sample based on the quotients of voltage and current. Of each spinning specification, 7 samples were tested.

Microscopic images of the cross section of spun filaments were obtained by means of the light microscope Zeiss Ultra Plus with Axio Imager M1 from Carl Zeiss AG (Jena/Oberkochen, Germany).

## 3. Results and Discussion

### 3.1. Spinning and Stretching Process

**Figure 3:**
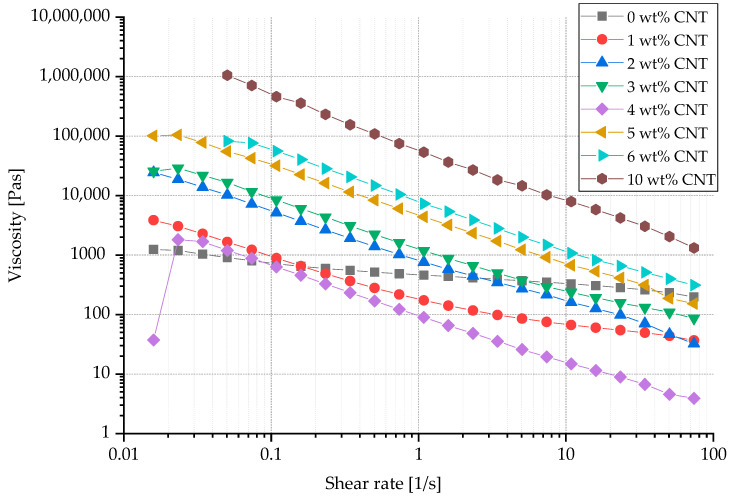
Viscosity of compounds with different carbon nanotube (CNT) contents, measured at a constant temperature of 200 °C.

The addition of CNTs to TPU led to significant inhomogeneities at the nano level, thus reducing spinnability. Pure TPU showed a slightly shear-thinning material behavior, which was significantly increased by the addition of CNTs. However, increasing the CNT content also resulted in a considerable rise in melt viscosity (see Figure 3) and decrease in stretchability. For example, once the CNT content was increased from 1 wt.% CNT to 5 wt.% CNT, a tenfold increase in viscosity was observed at shear rates of 1–10 s^−1^, which are particularly relevant for the melt spinning process.

As a result, these filaments must be pulled off at a significantly slower pace compared to pure TPU (10 times higher) in order to avoid fiber breakage. TPU–CNT compounds with 0 wt.% or 1 wt.% offer the potential to be spun at high winding speeds of up to 650 m/min, whereas compounds with 2 wt.% or more CNT cannot be spun at speeds exceeding 37.2 m/min.

Especially in the case of low draw ratios and highly elastic melt, draw resonance can occur. This phenomenon causes an unevenness in the filament diameter, which at worst can lead to filament breakage [26]. In the experiments presented in this paper, there was a sharp increase in unevenness at a CNT content above 5 wt.% and a drawing speed of less than 15 m/min; hence, the resulting filaments were considered almost unusable for the desired purpose.

Due to high viscosities, great pressures of over 100 bar occurred at the spinneret, especially when processing compounds with high CNT contents. The reduced draw ratio in combination with constant extruder speed led to increasing filament diameters at increased CNT content. Moreover, the volume and the length-related filament mass increased accordingly. However, greater mass and minimized extensibility caused the polymer melt to emerge from the spinneret, which was no longer able to bear the weight of the solidifying filaments.

### 3.2. Microscopic Analyses

**Figure 4:**
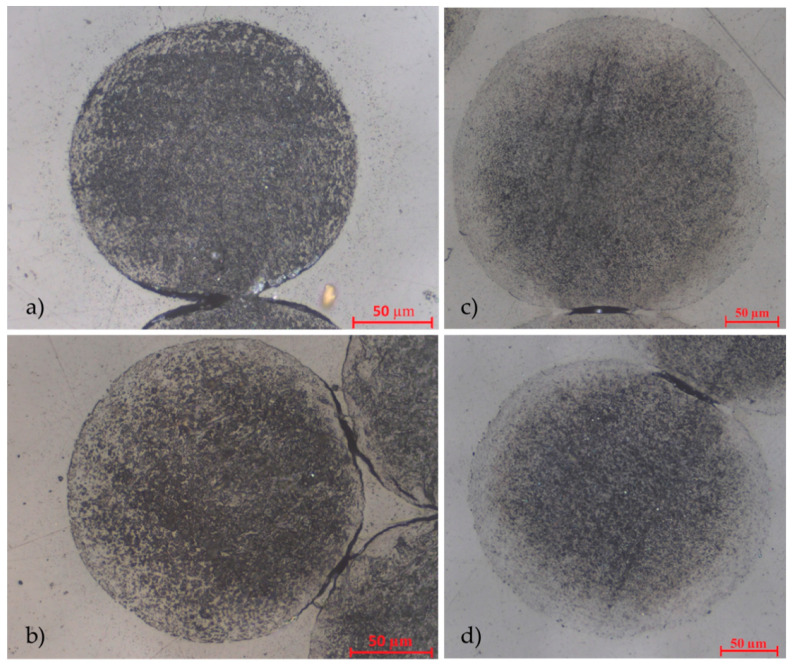
Cross-section of monocomponent filaments consisting of thermoplastic polyurethane (TPU) + CNT: (**a**) CNT content: 2 wt.%, spinning speed: 37.2 m/min; (**b**) CNT content: 4 wt.%, spinning speed: 30 m/min; (**c**) CNT content: 6 wt.%, spinning speed: 10 m/min; (**d**) CNT content: 6 wt.%, spinning speed: 17 m/min.

Figure 4 shows cross-sections of the melt spun filaments and the distribution of the CNTs. At a low CNT content of 2 wt.% (Figure 4a) CNTs are evenly distributed throughout the cross-section, whereas the CNT distribution becomes more inhomogeneous at a CNT content of 4 wt.% (Figure 4b). In the case of high CNT contents of 6 wt.% (Figure 4c,d), an outer sheath layer of CNT-poor TPU was formed during the spinning process. This means that although the TPU–CNT compounds were spun as monocomponents, a core–sheath structure was obtained. It can be assumed that the melt separated into components of high viscosity (high CNT content) and low viscosity (low CNT content) as it passed through the spinneret. During filament formation, the core was formed by high-viscosity melt, while low-viscosity melt formed into the sheath.

### 3.3. Stress–Strain Tests

All spun TPU–CNT filaments exhibited elongations at break of over 170 % and low Young’s moduli of less than 80 kPa. Table 1 lists the fineness, elongation at break, Young’s modulus, and electrical resistance of all filaments as a function of the CNT content and the winding speed.

The compounding of CNTs and TPU created a percolative system [44,45] with a percolation threshold between 3 and 4 wt.% CNT. The lowest achieved specific resistance was 110 ± 39 Ωcm. This value was obtained at an unstretched multifilament yarn with a CNT content of 5 wt.% and a spinning speed of 10 m/min. Under stretching load, the electrical resistances increase by up to one order of magnitude. This can be explained by the fact that the electrically conducting CNT particles are moved away from each other, so that percolation paths are interrupted. The lowest measured resistance value at 50 % relative elongation is 662 ± 221 Ωcm and was achieved at filaments with 5 wt.% CNT and 8 m/min spinning speed. At a relative elongation of 100 %, an electrical resistance of 2185 ± 608 Ωcm was recorded at 6 wt.% CNT and 10 m/min spinning speed.

In general, electrical resistance increased in the case of faster spinning speeds. This behavior can be explained by the fact that at high spinning speeds, the CNTs within the solidifying filament were pulled away from each other, thus interrupting percolation paths. However, electrical conductivity in the unstretched filaments was not further improved by adding more CNTs beyond 5 wt.%. In unloaded yarns, higher electrical resistances were measured at 6 wt.% CNT than at 5 wt.% CNT. It can be assumed that due to the increased CNT content, the tendency of the CNTs to agglomerate was also more pronounced. Hence, more clusters were formed within the filament without improving its electrical conductivity as a result of insufficient distribution of the CNTs within the TPU. However, at relative elongations of up to 100 %, some of these clusters may contribute to improving the filament’s electrical conductivity. The particles are pulled away from each other under mechanical load so that agglomeration are broken up, and more particles are available to build percolation paths. Therefore, filaments with 6 wt.% CNT offer lower electrical resistances at 100% relative elongation than filaments with 5 wt.% CNT.

**Figure 5:**
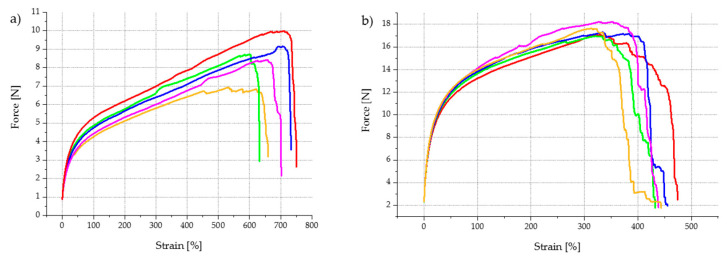
Stress–strain diagram of TPU multifilament yarns, melt spun at 15 m/min: (**a**) 97 wt.% TPU + 3 wt.% CNT; (**b**) 94 wt.% TPU + 6 wt.% CNT.

Figure 5 provides examples for stress–strain diagrams of multifilament yarns with 3 wt.% CNT and multifilament yarns with 6 wt.% CNT. Both compounds were spun at a take-off speed of 15 m/min.

It became evident that at higher CNT contents, the material failure of individual filaments occurred in a staggered manner. This suggests that the textile–physical properties of the filaments vary more widely among themselves than in compounds with a lower CNT content. At a CNT content of 6 wt.%, filaments began to break at an elongation of approx. 320%, whereas other individual filaments did not fail even at an elongation exceeding 440%. In contrast, at a CNT content of 3 wt.%, all filaments broke within a much smaller tensile force range. This confirms the assumption that the probability of CNT agglomeration increased significantly with increasing CNT content. Thus, the polymer network was more affected, causing local weak points to be generated in individual filaments and a staggered material failure to occur.

**Figure 6:**
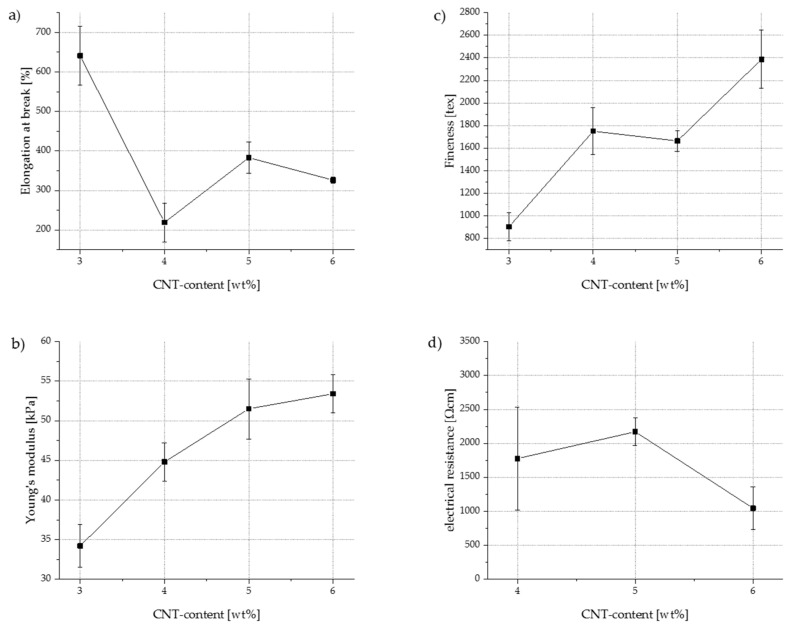
Mechanical and electrical properties of TPU–CNT multifilament yarns processed at a spinning speed of 15 m/min: (**a**) elongation at break as functions of CNT content in TPU; (**b**) Young’s modulus as functions of CNT content in TPU; (**c**) fineness as functions of CNT content in TPU; (**d**) electrical resistance as functions of CNT content in TPU.

Furthermore, it was observed that the maximum tensile strength of the filaments increased, whereas the elongation at break decreased with increasing CNT content. For a multifilament with 3 wt.% CNT, the average tensile strength was 8.65 N, and the average elongation at break was 641%. If the CNT content increased to 6 wt.%, the average tensile strength almost doubled to 17.4 N; simultaneously, the elongation at break almost halved to 326 %. Figure 6a represents the elongation at break as a function of the CNT content at a constant spinning speed of 15 m/min. It can also be seen that Young’s modulus (Figure 6b) and fineness (Figure 6c) increase with increasing CNT content, while elongation at break decreases. The data collected for electrical resistance (Figure 6d) suggest large standard deviations, especially at a CNT content of 4 wt.% (specific electrical resistance: 1777 ± 756 Ωcm); thus, further investigations are needed to increase reliability. For multifilament yarns containing less than 4 wt.% CNT, no electrical resistance could be measured, because it is beyond the measurable range. This indicates that the percolation threshold must lie between 3 wt.% CNT and 4 wt.% CNT.

**Figure 7:**
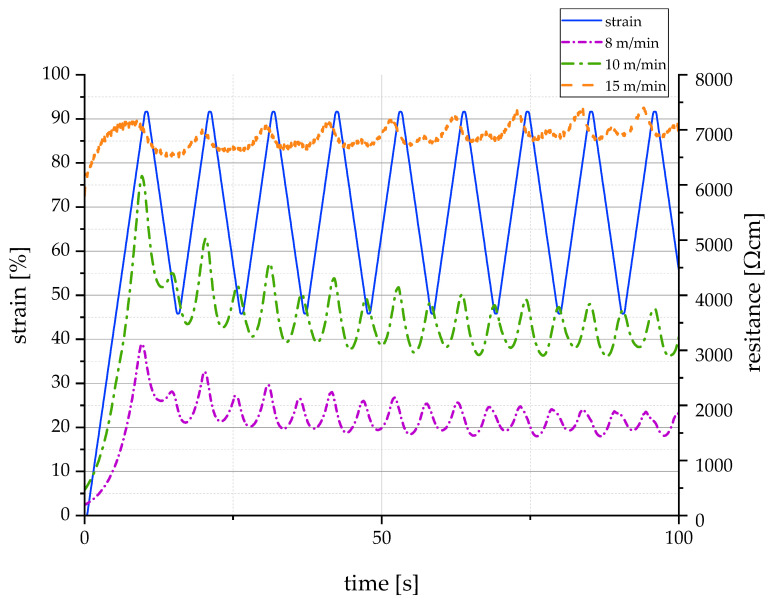
Correlation between mechanical elongation and electrical resistance for filament yarns containing 5 wt.% CNT and melt spun at different winding speeds.

Even the lowest value of electrical resistance was several orders of magnitude higher than the resistivity of fine copper wires ($1.7\times 10^{-6}$ Ωcm [46]); however, it was in the same range as electrically conductive liquid rubber (30–75 Ωcm [47,48]). Thus, CNT-filled TPU is suitable for a wide range of sensors and actuators, as its combination of high elasticity, electrical conductivity, and spinnability results in a completely new property profile. Figure 7 provides a first impression of the sensorial behavior of the melt spun fibers. The diagram shows the correlation between mechanical elongation and electrical resistance for three different filament yarns, each containing 5 wt.% CNT but spun at different winding speeds. It can be seen that the electrical resistance increases with increasing winding speed in the spinning process. Furthermore, the intermediate peaks (also known as shoulder phenomenon) are less pronounced with increasing winding speed. Nevertheless, there is no unambiguous correlation between mechanical elongation and electrical resistance in any specification. Further investigations will follow to determine the extent to which pretreatment of the filaments by means of mechanical pre-stretching or annealing has a positive influence on the sensorial behavior.

Due to the very high viscosity gradient in the liquid compound, a CNT-rich region was formed in the filament core, while a sheath of almost pure TPU surrounded this core (see Figure 4c,d). Thus, without the need for an additional work step or a bicomponent melt spinning process, a core–sheath filament with an electrically conductive filament core and an insulating sheath layer was created. This insulating layer offers advantages for many applications, for example, by minimizing the probability of undesirable short circuits in sensor networks. It is also worth mentioning that the sheath established a strong physical and chemical bond with the electrically conductive filament core, hence encouraging the assumption of high fatigue strength.

## 4. Conclusions

By adding CNTs to TPU, fibers that are elastic and electrically conductive can be melt spun. The resulting filaments exhibited a very high elongation at break while providing mechanical properties in the range of conventional elastic fibers and electrical conductivities in the range of electrically conductive liquid rubbers. Additionally, the fiber core established a highly favorable bond with the surrounding insulating layer of pure TPU. In future research projects, the insulating properties have to be determined more specifically and the surrounding sheath has to be thoroughly investigated in terms of potential conducting flaws. Thus, the newly developed TPU–CNT filaments are durable and highly stress resistant. This new class of electrically conductive, highly stretchable yarns offers a great potential for sensors (for example, as strain sensors, pressure sensors, and electrochemical sensors) and actuators (for example, in dielectric elastomer actuators). Furthermore, these yarns could be used in wearable smart textiles for energy harvesting, computing, and communication.

## Figures and Tables

**Table 1:** Mechanical and electrical properties of TPU–CNT filaments.

CNT Content in wt.%	Winding Speed in m/min	Fineness in Tex	Elongation at Break in %	Young’s Modulus in kPa	Specific Electrical Resistance in Ωcm	Specific Electrical Resistance in Ωcm at 50% Relative Elongation	Specific Electrical Resistance in Ωcm at 100% Relative Elongation
0	400.0	53 ± 2	717 ± 85	37.0 ± 4.4	> 2 × 10^5^	> 2 × 10^5^	> 2 × 10^5^
	650.0	50 ± 5	793 ± 84	21.8 ± 6.9	> 2 × 10^5^	> 2 × 10^5^	> 2 × 10^5^
1	600.0	33 ± 1	669 ± 60	48.9 ± 9.4	> 2 × 10^5^	> 2 × 10^5^	> 2 × 10^5^
	650.0	32 ± 1	518 ± 82	55.7 ± 14.1	> 2 × 10^5^	> 2 × 10^5^	> 2 × 10^5^
2	37.2	386 ± 74	294 ± 26	56.9 ± 6.4	> 2 × 10^5^	> 2 × 10^5^	> 2 × 10^5^
3	15.0	903 ± 123	641 ± 75	34.2 ± 2.7	> 2 × 10^5^	> 2 × 10^5^	> 2 × 10^5^
	25.0	637 ± 122	524 ± 57	52.7 ± 5.0	> 2 × 10^5^	> 2 × 10^5^	> 2 × 10^5^
	37.2	271 ± 83	177 ± 21	74.7 ± 6.9	> 2 × 10^5^	> 2 × 10^5^	> 2 × 10^5^
4	12.0	2102 ± 281	350 ± 44	27.2 ± 0.9	586 ± 411	3776 ± 482	12,909 ± 314
	15.0	1750 ± 208	219 ± 49	44.8 ± 2.4	1777 ± 756	9011 ± 2317	14,057 ±750
	20.0	1114 ± 133	505 ± 38	45.9 ± 2.6	4213 ± 975	12,401 ± 1343	9974 ± 274
	25.0	1149 ± 195	346 ± 18	41.4 ± 1.9	6668 ± 662	10,808 ± 1521	10,048 ± 344
	30.0	1211 ± 86	196 ± 32	29.9 ± 3.7	3904 ± 1553	6119 ± 1595	9942 ± 392
5	8.0	2314 ± 294	292 ± 23	63.0 ± 3.4	131 ± 48	663 ± 221	2571 ± 967
	10.0	2171 ± 422	400 ± 18	46.4 ± 5.7	110 ± 39	1429 ± 303	5243 ± 811
	15.0	1664 ± 92	383 ± 39	51.5 ± 3.8	2170 ± 201	17,441 ± 646	14,377 ± 296
6	10.0	2950 ± 207	339 ± 40	44.6 ± 1.8	151 ± 41	723 ± 156	2185 ± 608
	15.0	2387 ± 258	326 ± 8	53.4 ± 2.4	1045 ± 316	1024 ± 424	2337 ± 831
	17.0	1764 ± 217	293 ± 26	80.0 ± 3.0	2077 ± 405	2429 ± 295	10,234 ± 1202
	20.0	1672 ± 272	246 ± 28	72.8 ± 3.9	1717 ± 448	1814 ± 239	5980 ± 2631

## Data Availability

The data presented in this study are available on request from the corresponding author.
